# Extracts from an Address Delivered in the Royal College of Chemistry, London

**Published:** 1846-12

**Authors:** 


### ECLECTIC DEPARTMENT,

### AND SPIRIT OF THE MEDICAL PERIODICAL PRESS.

---

*Extracts from an Address delivered in the Royal College of Chemistry, London.* By John Gardener, M. D.

This address published in the London Lancet, sets forth in glowing terms the condition and prospect of the science of Chemistry. The following extract relating to the school of Giessen, or, in other words, the school of the illustrious Liebig, will, we think, be read with interest.—*Ed. Buffalo Journal.*

"The method of study adopted at Giessen having been successful, so many chemists of reputation in this country having studied there, and being accustomed to proclaim this fact in demanding public confidence, it became a matter of primary importance to ascertain wherein the peculiarities of that school consisted. I am able very distinctly to assign the causes of the pre-eminence of Giessen as a school of chemistry.

In the first place, the reputation of Professor Liebig would attract students, and the liberality of the Hessian government enables the Professor to charge fees sufficiently moderate to come within the means of young men from the manufacturing, trading, and professional classes, and to employ qualified assistants to take the burthen of teaching. Secondly, a system of study is pursued, which embraces an initiation into all the manipulations required in the practice of the science, keeps the student's attention constantly on the alert, sharpens his faculties of observation, obliges him to become acquainted with the principles and theories of chemistry, in order to draw correct inferences from the phenomena he creates, and, in one word, acts as a rigid mental dicipline, as well as a technical introduction to all the facts at present known. The chemical student there, must possess the preliminary qualifications of a speculative acquaintance with the science. He then begins a course of experiments with his own hands, which produce the actions or manifestations which are characteristic of every known substance, at least, of all which are of ordinary occurrence. The plan is contained in the little work, entitled, "Outlines of the Course of Qualitative Analysis followed in the Giessen Laboratory," which has lately been presented to the English public.

The student takes potassa, soda, ammonia, baryta, magnesia, lime, alumina, and all the metals and their oxides; and with each he mixes the re-agents, which have, by the consent of chemists, been established as infallibly manifesting their peculiar reaction, and thus detecting with certainty their presence or absence in any given compound. In these experiments he has the constant assistance of a qualified teacher, to guard him against the use of too little or too great proportions of water, spirit, or other solvents—too great or too little heat—to show him the best manner of manipulating, and to assist him in interpreting the results. These results are certain appearances, mostly addressed to the eye; and, consequently, when spoken of as colors more or less vivid—precipitates more or less distinct, &c.,—form subjects, beyond any other in the whole range of human knowledge requiring the aid of an experienced teacher to define. The experiments thus to be made amount to many hundreds. When the student becomes familiar with all the phenomena produced by his re-agents with these bodies, and is able to assign the cause—to give the rationale of the reactions—he begins the analysis of unknown substances—that is, of substances unknown to himself, but which are prepared by the professor, and arranged in order, so as to lead the student, by slow and safe steps, from simple to complex cases. This they call going through the bottles. About twenty liquids, containing each only one base, are first taken; then, as many with one acid and one base, subsequently, two or more bases, two or more acids, and so on, until the more complex cases are reached; and, in the end, all the inorganic bases, and a number of acids in admixture, are analysed, to try the student's skill and memory.

So important is this method of study felt to be, that even many men, with a high reputation for scientific knowledge, as soon as they have looked closely at its effects, have at Giessen submitted to go through the drudgery of this systematic course.

When perfectly familiar with qualitative analysis—that is, when possessed of the power, imparted in this course, of detecting the presence or absence of every element in any possible form of mixture or compound—the student is taught by a similar system, to separate and estimate the quantities of every individual constituent: this is quantitative analysis; afterwards, the preparation of organic matter, and its analysis by combustion, becomes comparatively an easy step.

The constant presence, the incessant stimulus, and immediate aid of a qualified instructor, is the corner-stone of this system. It has a parallel only in the custom, at our great English universities, of working with a private tutor when the student aspires to honors in classics or mathematics. Every difficulty, as it is met with, is explained; every fact for which the student feels at a loss, is imparted; and his daily labor is definitely marked by the point reached in his onward progress."

We quote his remarks on the subject of organic chemistry—a subject of deep interest to the Physiologist, the Pathologist, and the Practical Physician.

"The point, however, to which I wish to direct your special attention, in reference to our anticipations of advancing the science, is organic chemistry. The apparatus for organic combustion, the great invention of Professor Liebig, is already almost daily in use in our laboratory. By its means, as you know, the analysis of matter belonging to the animal and vegetable kingdoms—the product, in some form or other, of the vital force—is effected with greater ease and equal precision with the analysis of a soil or a mineral. The rapidity with which discoveries are being made, and the immense extent of the science, render it almost impossible, except for the professed chemist, to follow and to appreciate its importance. Every now and then, however, some fact is made known, having an immediate application to practical purposes, and indicating how vast a field of inquiry exists in organic nature. Within the last few days, Professor Liebig has announced to us that a residue left in the manufacture of sulphate of quinine, is the pure alkaloid itself, merely obscured by its form. The discovery of quinine by Pellatier, in 1820, revolutionized pharmacy. The active principles of the most nauseous drugs can now be isolated from the accompaniments of woody fibre and other inert matters, and exhibited in a concentrated form. This is a blessing, which only those who have had to swallow bark in substance can fully understand. During the twenty-six years in which sulphate of quinine has been manufactured, a considerable portion, every year, of the pure alkaloid has been laid aside—and, consequently, a considerable amount has accumulated. Professor Liebig's discovery will not only bring this into use, but it will enable the practitioner of medicine to prescribe the pure alkaloid, and to combine it at pleasure with vegetable or other acids, and thus to obviate the objections which are found in practice to lie against the sulphate of quinine, placing at command quinine and a variety of its salts at a price which will not preclude the poorest from its benefit.

A great proportion of the vegetable kingdom still requires to be chemically investigated; of the materials which constitute the animal body, every one, without exception, awaits a new analysis and investigation. Not many months since, Professor Redtenbacher, of Prague, found that taurine, a constituent of bile, which acts a most important part in the animal economy, contains sulphur to the extent of 26 per cent. This element has been previously overlooked, in consequence of what must be considered a defect in the method of analysis by combustion; in that practice the oxygen of the analysed body being estimated by the amount of loss, the equivalent of sulphur being precisely double that of oxygen, its presence did not disturb the calculation of the results. Professor Gregory has remarked, that this observation of Redtenbacher will probably turn out, in its consequences, one of the most important yet made in animal chemistry; and it cannot be doubted, that although our views must be changed in many points in consequence of it, our knowledge will be extended and rendered more precise and more capable of direct application to physiology and pathology. Already Professor Liebig has found reason to believe that Mulder's protein and oxides of protein exist only in imagination, as sulphur remains combined with the other elements after fibrin and albumen are subjected to Mulder's processes. To extend this inquiry into all the materials of the animal tissues, requires many skilful hands and much labor.

This branch of the science is occupying the attention of very many students on the Continent, and is undergoing a rapid development. From the masses of figures and calculations, now accumulating, representing analyses, we may anticipate many practical results of vast importance, to emerge.

For my own part, I entertain the most sanguine hopes that the diseases most fatal to mankind will be elucidated, and, perhaps, wholly prevented, by pursuing this path of chemical inquiry."

